# Association between NF-*κ*B Activation in Peripheral Blood Mononuclear Cells and Late Skin and Subcutaneous Fibrosis following Radiotherapy

**DOI:** 10.1155/2020/2957818

**Published:** 2020-07-21

**Authors:** Yapin Su, Yuyu Zhang, Meiting Sun, Wenhui Liu, Fengli Pei, Fujun Han

**Affiliations:** ^1^Cancer Center, The First Hospital of Jilin University, 71 Xinmin Ave, Changchun, China; ^2^Department of Radiation Oncology, The First Hospital of Jilin University, 71 Xinmin Ave, Changchun, China

## Abstract

**Background:**

This study aimed at evaluating the association between the speed of nuclear factor-kappa B (NF-*κ*B) activation in peripheral blood mononuclear cells (PBMCs) and late skin and subcutaneous fibrosis in patients with head and neck squamous cell carcinoma (HNSCC) after radiotherapy.

**Methods:**

The speed of NF-*κ*B activation was represented by the nuclear p65 expression ratio before and after irradiation. The optimal time point to measure the ratio was determined by Western blot in the PBMCs from healthy outpatients ranging from 0 to 12 hours after ex vivo irradiation. We recruited patients with HNSCC who had received ratiotherapy and who were under regular follow-up care. We assessed the association between the risk of developing ≥grade 2 late fibrosis and the nuclear p65 expression ratio in the PBMCs after ex vivo irradiation in these patients.

**Results:**

The maximum nuclear p65 ratio was observed at 1 hour after ex vivo irradiation in the PBMCs from the healthy outpatients. The speed of NF-*κ*B activation was then represented by the nuclear p65 ratio in the PBMCs before and 1 hour after ex vivo irradiation. A total of 200 patients with HNSCC were recruited, 32.50% (*n* = 65) of which presented with ≥grade 2 late fibrosis. There was a significant association between the speed of NF-*κ*B activation in the PBMCs and an increased risk of developing ≥grade 2 late fibrosis in these patients (*P* = 0.004). Subgroup analysis suggested that this finding was independent of the known clinical characteristics.

**Conclusions:**

The speed of NF-*κ*B activation might be a potential predictor of late toxicity in cancer patients after radiotherapy. Prospective studies are needed for validation.

## 1. Background

As a major treatment, radiotherapy is delivered for more than 50% of cancer patients at some time during the course of their disease [1]. Unavoidably, radiotherapy induces a wide spectrum of toxicity in a variety of tissues. The toxicity is classified as acute and late based on the time of onset. Unlike acute toxicity, which is recoverable and temporal, late toxicity usually cannot be halted and progress with time [2], resulting in a substantial decrease in quality of life. The severity of late toxicity varies markedly between individual patients even for those who had received similar or identical radiotherapy protocols [3]. This patient-related variability in late toxicity is considered to be mainly determined by genetic factors [4–6]. However, in spite of extensive studies, no genetic factors have been identified that are feasible for clinical use to predict the occurrence and severity of late toxicity [7, 8].

In response to ionizing radiation, a number of signal transduction pathways are activated, including apoptosis, immune response, inflammation, differentiation, proliferation, and cell survival. Nuclear factor-kappa B (NF-*κ*B) is a convergence of these pathways [9, 10]. NF-*κ*B is homodimers or heterodimers of the Rel family members, including NF-*κ*B1 (p50), NF-*κ*B2 (p52), c-Rel, RelA (p65), and RelB [11]. The most abundant form of NF-*κ*B is the heterodimer of p65-50 [12]. Inactive NF-*κ*B is mainly cytoplasmically localized. Upon activation, NF-*κ*B migrates into the nucleus, resulting in transcription of target genes [13]. Therefore, nuclear p65 is a marker for activated NF-*κ*B, and nuclear p65 expression is feasible to represent the degree of activation of NF-*κ*B signalling pathway.

There are some studies showing an association between NF-*κ*B activation and radiation-induced toxicity. Haase et al. found a marked and continuous increase in NF-*κ*B activation in the rat lung over 6 months after ionizing irradiation [14]. The elevation of NF-*κ*B activation was consistently observed in the damaged tissue by independent studies: Zhang et al.'s radiation-induced lung injury in rats [15] and Ye et al.'s radiation-induced heart injury in rats [16]. In addition, NF-*κ*B inhibition by p65 depletion can decrease irradiation-induced lung toxicity in a radiation-induced pulmonary fibrosis mouse model [17].

Kinetics of p65 activation in peripheral blood mononuclear cells (PBMCs) can be quantified, and the association with inflammation and cytokines has been investigated [18–20]. Skin and subcutaneous tissue are the most frequently affected organs by late radiation toxicity, commonly represented as fibrosis, atrophy, xerostomia, and telangiectasia. Of which, fibrosis is frequently observed in patients with head and neck squamous cell carcinoma (HNSCC) after radiotherapy. Here, we applied Western blot to quantify the nuclear p65 expression in the PBMCs from patients with HNSCC after ex vivo irradiation. The nuclear p65 expression ratio before and after irradiation, a representative of the speed of NF-*κ*B activation, was then correlated with late skin and subcutaneous fibrosis developed by these patients.

## 2. Methods and Materials

### 2.1. Patients

Between January 2017 and July 2019, patients with HNSCC under regular follow-up care were recruited. The eligibility criteria were as follows: patients must have received curative radiotherapy, curative radiotherapy with concurrent chemotherapy, or curative surgery plus adjuvant radiotherapy with or without concurrent chemotherapy, for at least 2 years; patients were recurrence free at the time of recruitment. Data on the tumor and its treatments were collected from patient records, and personal information was gathered by self-administered questionnaires. All participants were required free from severe diabetes mellitus, uncontrollable hypertension, and other severe diseases. Controls were randomly selected from the healthy outpatients aged between 18 and 75 in the same consulting room during the same period, and those with history of cancers were not eligible.

### 2.2. Assessments of Late Skin and Subcutaneous Fibrosis

Late radiation-induced skin and subcutaneous fibrosis was measured between January 2017 and July 2019 on outpatient follow-up. Fibrosis was graded according to the Radiation Therapy Oncology Group (RTOG) and the European Organization for Research and Treatment of Cancer (EORTC) system [21] by 2 oncologists. Disagreements were resolved by discussion between the oncologists with arbitration by a third reviewer when necessary. The oncologists were blinded to the operation of the experiments so as not to bias the evaluation.

### 2.3. PBMC Sampling

The PBMCs were separated following the procedure as previously reported [22]. Ten mL peripheral blood was taken from each patient and was anticoagulated with heparin in tubes. Lymphocyte separation medium (3 mL; cat. no. 1077; Sigma, China) was pipetted to a 15 mL centrifuge tubes, and 3 mL of peripheral blood was carefully overlaid on top of the lymphocyte separation medium and centrifuged at 550 g for 25 minutes at 20°C. PBMCs were collected from the lymphocyte separation medium interphase and were washed twice in phosphate-buffered saline (PBS, pH 7.4); these were plated in fresh culture medium (Human T Cell Culture Medium; cat. no. 1502; Cell Science Inc., Japan). The number of PBMCs obtained from 10 mL of blood ranged from 20 × 10^6^ to 30 × 10^6^ cells.

### 2.4. Nuclear Protein Extraction and Western Blot

Nuclear proteins were extracted from PBMC cells using nucleoprotein kit (cat. no. p5130; NCM Biotech, China) following the manufacturer's instructions. The protein concentration was determined by BCA protein assay kit (cat. no. P0010S; Beyotime, China). Any contamination of nuclear proteins in cytoplasmic fractions and vice versa was monitored by Western blot with antibodies to lysosomal-associated membrane protein 1 (LAMP1) (cytoplasmic marker; dilution 1 *μ*g/ml; cat. no. ab24170; Abcam, USA) and to histone H3 (nuclear marker; dilution 1 *μ*g/ml; cat. no. ab18521; Abcam, USA) [23].

Cell lysates were separated on 10% SDS-polyacrylamide gels and transferred to PVDF membrane. The membranes were blocked with TBST buffer containing 5% powered milk at room temperature, and then were probed overnight at 4°C with primary anti-p65 (dilution 1 : 500; cat. no. MAB3026; Merck, Germany). The membranes were then incubated with horseradish peroxidase-linked secondary antibodies (dilution 1 : 1000; cat. no. A0208; Beyotime, China) and were developed using the ECL chemiluminescent reagents (cat. no. P10100A and P10100B; NCM Biotech, China). Next, each blot was stripped, and the membrane was reprobed with a primary antihistone H3 antibody.

Blot images were captured using a chemiScope imaging system (ChemiScope 5300, CLINX, China). Band densitometry was obtained using Image J [24]. For each patient, the gray value of the histone H3 band was normalized to the maximum one, which was defined as a value of 1. The density of the p65 band was adjusted by dividing its value to the corresponding normalized histone H3 value.

### 2.5. Choice of Time Points

We used Western blot analysis to assess nuclear p65 expression in the PBMCs from the controls from 0 to 12 hours after 2-Gy ex vivo irradiation. We aimed at identifying the time point when maximum nuclear p65 expression occurred in the PBMCs after ex vivo irradiation.

### 2.6. Immunofluorescence and Automated Image Analysis

The immunofluorescence staining was prepared following the procedure as previously reported [22, 25]. Briefly, isolated PBMCs were fixed in 4% (*v*/*v*) paraformaldehyde in PBS for 20 min at room temperature. Next, they were cytospin on microscopy slides for 10 minutes at 3000 rpm, and then permeabilized with 0.5% Triton X-100 in PBS for 10 min at room temperature. Next, the cells were blocked in PBS/5% w/v BSA, pH 7.4, for 20 minutes, and then incubated for 1 hr at room temperature with primary anti-p65 (dilution 1 : 500; cat. no. MAB3026; Merck, Germany) antibody. After washed with PBS, the cells were incubated with FITC-labeled secondary antibody (dilution 1 : 500; cat. no. A0428; Beyotime, China) for 45 min at room temperature. Last, cells were stained with DAPI (dilution 1 : 5000; cat. no. C1002; Beyotime, China) for 10 min. Coverslips were mounted in Mowiol mounting medium. Image capturing was performed with an inverted fluorescence microscope (IX51, OLYMPUS, Japan) with 40x objective and a digital camera (DP72, OLYMPUS, Japan).

**Figure 1:**
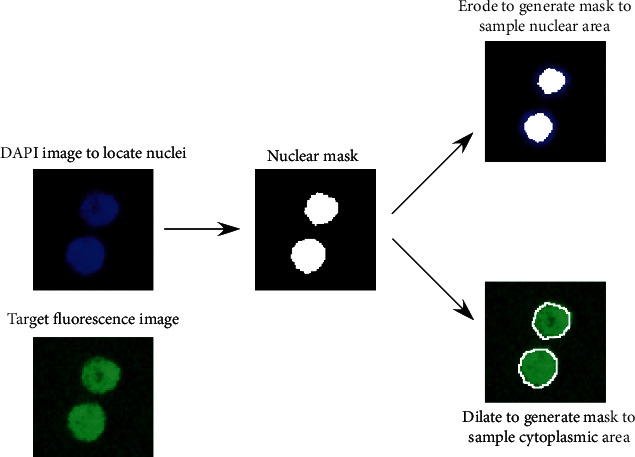
Automated image analysis of nuclear: cytoplasmic ratio.

The automated image analysis was coded by Matlab (R2015a, The MathWorks, Natick, MA, USA) as indicated in Figure 1. A nuclear mask was acquired by segmentation of the DAPI-stained images according to Han et al.'s methods [25, 26]. To reduce cytoplasmic contamination, the nuclear mask was eroded, and the eroded mask was applied to quantify target channel fluorescence within the nucleus. The nuclear mask was then dilated to cover the cytoplasmic region without going outside the cell boundary. A ring mask that covers the cytoplasmic region was created by removing the original nuclear region from this dilated mask. The nuclear: cytoplasmic ratio was calculated as the median nuclear intensity divided by the median cytoplasmic ring intensity on a per cell basis.

### 2.7. Statistical Analysis

All statistical analyses were performed using R, version 3.5.2. Chi-squared or Fisher's exact test was used to test differences in covariates in patients with ≥grade 2 and those with <grade 2 late skin and subcutaneous fibrosis. The Wilcoxon rank-sum test was used to compare the p65 expression difference between patients with ≥grade 2 and those with <grade 2 late skin and subcutaneous fibrosis. The difference was also assessed by using a multiple linear regression model adjusted for age, sex, smoking history, alcohol consumption, TNM stage, chemotherapy treatment, and surgery. Subgroup analyses were conducted according to clinical characteristics, including age (<65 years *vs.* ≥65 years), sex (men *vs.* women), smoking history (never *vs.* ever), alcohol consumption (never *vs.* ever), TNM stage (I-II *vs.* III-IVa-b), chemotherapy treatment (yes *vs.* no), and surgery (yes *vs.* no). All statistical tests were two-sided. A *P* value of <0.05 was used for a statistical significance.

## 3. Results

### 3.1. Patient Characteristics

Twelve controls were eligible, including 7 males (58%) and 5 females (42%), and median age was 60 years (range, 35 to 74 years). A total of 211 patients were recruited, of which 11 patients were excluded from subsequent analyses: PBMCs isolation failed in 2 patients; protein extraction was inadequate in 4 patients; and Western blot failed in 5 patients. Among the remaining patients (*n* = 200), 65 (32.50%) patients presented with ≥grade 2 late fibrosis (Table 1). At the time of late toxicity assessment at the outpatient visits, median follow-up from the last day of radiotherapy was 3.2 years (range: 2.0–6.8 years).

Table 2 lists the demographic and clinical features of the patients presented with ≥grade 2 late fibrosis and those presented with <grade 2 late fibrosis. The features balanced between the two groups included age at diagnosis, gender, primary site, radiation technique, chemotherapy (yes/no), and surgery (yes/no). In contrast, a significant difference was observed in smoking history (*P* = 0.020), alcohol consumption (*P* = 0.041), and tumor stage at diagnosis (*P* = 0.026).

### 3.2. Maximum Speed of NF-*κ*B Nuclear Accumulation

**Figure 2:**
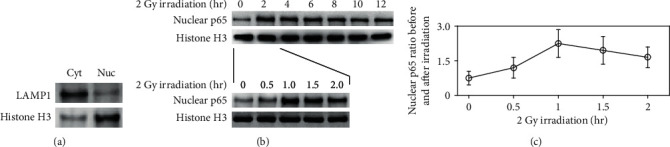
Nuclear NF-*κ*B p65 expression at various time points in the PBMCs after ex vivo irradiation. Cyt: cytoplasm; Nuc: nuclei. The nuclear p65 expression in the PBMCs was measured from 0 to 12 hours in a time interval of 2 hours after 2-Gy ex vivo irradiation. All samples showed a maximum nuclear p65 expression at 2 hours. The measurement was then repeated from 0 to 2 hours in a time interval of 0.5 hours, and all samples (*n* = 3) showed a maximum at 1 hour. Any contamination of nuclear proteins in cytoplasmic fractions and vice versa was verified by Western blot with anti-LAMP1 (cytoplasmic marker) and antihistone H3 (nuclear marker). This experiment was repeated 3 times, and consistent results were obtained. (a) Representative Western blot bands of the purity verification of the nuclear protein extraction. (b) Representative Western blot bands of the nuclear p65 translocation after the irradiation. (c) The nuclear p65 expression ratio before and after the irradiation at the time points (c).

The nuclear p65 expression in the PBMCs from 3 controls was initially measured from 0 to 12 hours in a time interval of 2 hours after 2-Gy ex vivo irradiation. Time-related patterns of the nuclear p65 expression were similar among these samples. Almost all samples showed a maximum nuclear p65 expression at 2 hours after irradiation. We then repeated the measurement from 0 to 2 hours in a time interval of 0.5 hours, and all samples (*n* = 3) showed a maximum nuclear p65 expression at 1 hour. This experiment was then repeated 3 times, and consistent results were obtained. Representative Western blot bands and distribution of the nuclear p65 expression ratio before and after irradiation are shown in Figures 2(a)–2(c). We therefore considered the appropriate time points to assess the nuclear p65 expression ratio would be 0 hour and 1 hour after irradiation.

### 3.3. Immunofluorescence and Automated Image Analysis

**Figure 3:**
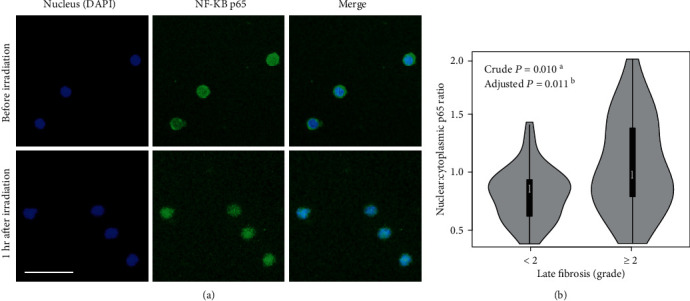
Distribution of NF-*κ*B p65 by immunofluorescence before and after irradiation in PBMCs from HNSCC patients. NF-*κ*B p65 immunofluorescence staining was successfully performed in the PBMCs in 12 patients. Shown are representative images (a). Arrows show p65 in the PBMCs, from dense staining in the cytoplasm before irradiation to dense staining in the nuclei at 1 hour after ex vivo irradiation with 2 Gy. Scale bars: 50 *μ*m. (b) Automate image analysis of nuclear: cytoplasmic p65 expression ratio of irradiated (1 hour after irradiation with 2 Gy) PMBCs in patients with ≥2 versus those with <2 grade late skin and subcutaneous fibrosis. ^a^Crude *P* value from the Wilcoxon rank-sum test. ^b^Adjusted *P* value from the multiple linear regression model adjusted for age, sex, smoking history, alcohol consumption, TNM stage, chemotherapy treatment, and surgery.

Immunofluorescence microscopy was applied to assess kinetics of p65 in irradiated PMBCs for 100 patients. There was a substantial loss of cells during the immunofluorescence staining process in 23 patients' samples, leaving 77 patients eligible for further analysis. The PBMCs showed mild nuclear and condense cytoplasmic staining before irradiation. One hour after exposure to irradiation (2 Gy), a significant increase of p65 in the nuclei was evident. Shown are representative images (Figure 3(a)).

Automated image analysis showed that the median p65 nuclear: cytoplasmic ratio was 0.80 (range: 0.35–1.38) for <2 grade late fibrosis, and was 0.92 (range: 0.36–1.91) for ≥2 grade late fibrosis. The nuclear p65 expression ratio was statistically different (crude *P* = 0.010) by the Wilcoxon rank-sum test between patients with ≥2 grade and those with <2 grade late skin and subcutaneous fibrosis (Figure 3(b)). The difference (adjusted *P* = 0.011) remained by multiple linear regression model adjusted for age, sex, smoking history, alcohol consumption, TNM stage, chemotherapy treatment, and surgery.

The significant loss of cells in 23% samples made this method infeasible in practical applications, and we terminated this experiment after the first 100 patients were finished. Due to the small sample size, subgroup analyses were not conducted.

### 3.4. NF-*κ*B Nuclear Accumulation Speed and Late Toxicity

**Figure 4:**
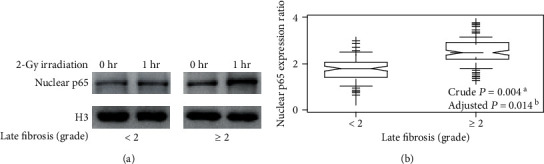
Late radiation-induced fibrosis in patients and NF-*κ*B p65 nuclear accumulations in their PMBCs. The purity of the nuclear extracts from contamination of the cytoplasmic fractions is shown by representative Western blot bands (Figure 1(a)). (a) Representative Western blot bands reflect characteristic differences in band densities between nuclear p65 of irradiated (1 hour after irradiation with 2 Gy) and nonirradiated PMBCs in 2 patients. (b) Categorical analysis of nuclear p65 expression ratio of the irradiated to the nonirradiated PMBCs in patients with ≥2 versus those with <2 grade late skin and subcutaneous fibrosis. ^a^Crude *P* value from the Wilcoxon rank-sum test. ^b^Adjusted *P* value from the multiple linear regression model adjusted for age, sex, smoking history, alcohol consumption, TNM stage, chemotherapy treatment, and surgery.

The nuclear p65 expression was assessed with Western blot analysis at 0 hour in nonirradiated and 1 hour in irradiated PMBCs for all patients. The median nuclear p65 ratio was 2.04 (range: 1.09–3.57) for <2 grade late fibrosis and was 2.51 (range: 1.25–3.76) for ≥2 grade late fibrosis. Shown are representative images (Figure 4(a)). The nuclear p65 expression ratio was statistically different between patients with ≥2 grade and those with <2 grade late skin and subcutaneous fibrosis (crude *P* = 0.004 by the Wilcoxon rank-sum test and adjusted *P* = 0.014 by multiple linear regression model adjusted for the variables, Figure 4(b)). Subgroup analyses according to clinical characteristics yielded roughly the same tendency in agreement with the main results (Table 3).

## 4. Discussion

In this study, we analyzed the association of NF-*κ*B activation with the development of radiation-induced late toxicity in patients with HNSCC treated with radiotherapy. Our findings suggested that the risk of radiation-induced late skin and subcutaneous fibrosis was significantly increased in patients who had a higher speed of NF-*κ*B nuclear accumulation in their PBMCs after ex vivo irradiation. This finding appeared to be independent of age, sex, primary site, tumor stage, surgery, or chemotherapy.

We observed an association between an early NF-*κ*B activation and late tissue toxicity in cancer patients after radiotherapy. It is of great convenience for oncologists to predict the occurrence and severity of late toxicity beforehand by an easy essay. PBMCs can be easily obtained from the peripheral blood of patients. The NF-*κ*B activation in the PBMCs after irradiation is apparently an early response, and PBMCs were also considered as an early responding tissue [22]. Surprisingly, there was an association between NF-*κ*B activation and late fibrosis. Actually, this was analogous to the radiosensitivity of patients with genetic syndromes, such as ataxia-telangiectasia and Nijmegen breakage syndromes [27, 28], suggesting a general increased radiosensitivity across various cell types and across various temporal extents. Ionizing radiation-induced DNA damage leads to cellular responses including DNA damage response, apoptosis, immune response, inflammation, and cell-cycle arrest. Consequently, genetic variability in these signalling pathways could influence radiosensitivity, and in turn could be associated with acute and late toxicity [29–32]. As a central element in these pathways, it is plausible that NF-*κ*B activation is associated with radiation-induced toxicity. Indeed, continuous NF-*κ*B activation was associated with radiation-induced toxicity in rat models [14–17]. It is unknown whether the speed of NF-*κ*B activation correlates with the lasting time of NF-*κ*B activation leading to late toxicity. To minimize the potential source of confusion due to long time culture of PBMCs, we did not assess the association of late toxicity with the lasting time of the NF-*κ*B activation in the present work. The relationship between the speed of NF-*κ*B activation, the lasting time of NF-*κ*B activation, and late toxicity needs further investigation.

Our finding of an association between NF-*κ*B activation and late radiation-induced fibrosis added to current knowledge of associations between molecular radiosensitivity and late tissue toxicity. Late fibrosis is featured by a large deposition of extracellular matrix molecules (e.g., collagens, glycosaminoglycans, and proteoglycans) and an excessive of fibroblast proliferation [33, 34]. The survival fraction of cultured fibroblast at 2 Gy was reported to be associated with late fibrosis in the same patients [35, 36]. In addition, genetic studies identified an association between late radiation-induced fibrosis and polymorphisms in the ATM, TGFB, ERCC1, and ERCC5 genes for cancer patients who had been treated with radiotherapy [37–40]. The development of late fibrosis involves a cytokine cascade and a variety of different pathological processes, of which fibroblast, genetic polymorphisms, or NF-*κ*B is only one of the many elements. Until date, a large number of studies had been conducted to identify cellular and molecular factors that might influence the occurrence and severity of late toxicity. Most of these studies investigated only a single element, whereas the relationship among these elements has not been investigated. For example, it is unknown whether fibroblast radiation sensitivity correlates with polymorphisms in these genes that lead to late toxicity. These studies had evoked interest, but no general agreement has been reached regarding any of these factors that could be sufficiently accurate for the prediction of radiation-induced toxicity for clinical use [7, 8, 41]. Future studies focus on the relationship among these elements might better clarify the mechanism of late toxicity, and eventually develop biomarkers for clinical use.

Limitations in this work included the retrospective nature and heterogeneity in the treatment protocols. In addition, radiotherapy modalities were not assessed in detail due to the lack of some information and small sample size in some treatment protocol. As an explorative study, we tried all our ways to minimize any confusion. For example, we adopted a single primary endpoint, late fibrosis, because the underlying causes of late toxicity in different tissue and in different forms (e.g., fibrosis, atrophy, or telangiectasia) were considered to be different. To minimize technical confusions, we used a method as simple as possible—the nuclear p65 expression ratio in the PBMCs before and 1 hour after ex vivo irradiation. Due to these limitations, our findings should be interpreted with caution.

In conclusion, our analysis found that a higher speed of nuclear p65 accumulation in the PMBCs after irradiation was associated with an increased risk of developing ≥2 grade late skin and subcutaneous fibrosis in patients with HNSCC after radiotherapy. This finding adds to the growing knowledge about translation of molecular radiosensitivity into practical radiological protection, but how NF-*κ*B activation related to fibrosis remains unknown. Further studies will be required to understand this association.

## Figures and Tables

**Table 1:** Late radiation-induced skin and subcutaneous fibrosis.

RTOG/EORTC	No.	%
Grade 0	52	26.00
Grade 1	83	41.50
Grade 2	47	23.50
Grade 3	17	8.50
Grade 4	1	0.50

EORTC: European Organization for Research and Treatment of Cancer; RTOG: Radiation Therapy Oncology Group.

**Table 2:** Patients and disease characteristics stratified by grade of late fibrosis.

Variables	Grade <2		Grade ≥2		*P* value
	No. (*n* = 135)	%	No. (*n* = 65)	%	
Age at diagnosis					0.383
<64	100	74.07	52	80.00	
≥65	35	25.93	13	20.00	
Gender					0.584
Male	105	77.78	53	81.54	
Female	30	22.22	12	18.46	
Smoking history					0.020
Yes	92	68.15	33	50.77	
No	43	31.85	32	49.23	
Alcohol consumption					0.041
Yes	106	78.52	42	64.62	
No	29	21.48	23	35.38	
Primary site					0.119
Oral cavity	23	17.04	8	12.31	
Oropharynx	15	11.11	3	4.62	
Hypopharynx	15	11.11	13	20.00	
Larynx	42	31.11	28	43.08	
Nasopharynx	38	28.15	13	20.00	
Unknown	2	1.48	0	0.00	
Tumor stage at diagnosis^a^					0.026
I	33	24.44	8	12.31	
II	25	18.52	19	29.23	
III	59	43.7	22	33.85	
IV_a_-IV_b_	15	11.11	15	23.08	
Unknown	3	2.22	1	1.54	
Radiation technique					0.976
3D-CRT	41	30.37	20	30.77	
IMRT	77	57.04	36	55.38	
Unknown	17	12.59	9	13.85	
Chemotherapy					0.999
Yes	32	23.7	16	24.62	
No	103	76.3	49	75.38	
Surgery					0.764
Yes	70	51.85	32	49.23	
No	65	48.15	33	50.77	

^a^According to the American Joint Committee on Cancer TNM staging system for head and neck cancers (7th ed., 2010) staging criteria.

**Table 3:** NF-*κ*B p65 nuclear accumulations in PMBCs according to grade of late fibrosis and patient subgroups.

Variables^a^	Grade <2 late fibrosis		Grade ≥2 late fibrosis		Crude *P* value^b^	Adjusted *P* value^c^
	Median	**IQR**	Median	**IQR**		
Age						
<65	2.08	1.86-2.59	2.40	2.00-3.02	0.023	0.070
≥65	1.95	1.67-2.56	2.64	1.85-2.77	0.171	0.112
Gender						
Male	2.08	1.86-2.63	2.51	1.88-3.02	0.062	0.089
Female	1.90	1.69-2.15	2.34	2.07-2.77	0.008	0.007
Smoking						
Yes	2.03	1.77-2.54	2.52	2.08-2.86	0.004	0.003
No	2.08	1.90-2.77	2.36	1.96-3.02	0.444	0.590
Alcohol consumption						
Yes	2.02	1.79-2.55	2.56	2.05-2.93	0.002	0.013
No	2.14	1.88-2.66	2.29	1.93-3.02	0.846	0.688
Primary site						
Nasopharynx	2.23	1.87-2.66	2.81	2.17-3.03	0.150	0.299
Nonnasopharynx	2.02	1.78-2.54	2.40	1.89-2.91	0.010	0.027
TNM stage						
I-II	2.03	1.77-2.51	2.20	1.93-2.73	0.069	0.063
III-IVa-b	2.12	1.85-2.65	2.72	2.06-3.03	0.014	0.030
Radiotherapy						
3D-CRT	1.94	1.66-2.34	2.64	2.08-3.01	0.026	0.026
IMRT	2.08	1.86-2.63	2.37	1.94-3.02	0.134	0.308
Chemotherapy						
Yes	1.93	1.64-2.25	2.10	1.87-2.79	0.088	0.189
No	2.08	1.87-2.62	2.53	2.01-2.95	0.014	0.025
Surgery						
Yes	2.14	1.87-2.62	2.68	1.88-3.02	0.079	0.109
No	1.99	1.69-2.54	2.30	2.01-2.86	0.014	0.074

IQR: interquartile range. NF-*κ*B p65 nuclear accumulations were represented by nuclear p65 expression ratio of irradiated (1 hour after irradiation with 2 Gy) to nonirradiated PMBCs. ^a^Sample size in each subgroup was listed in Table 1. ^b^Crude *P* value from the Wilcoxon rank-sum test. ^c^Adjusted *P* value from the multiple linear regression model adjusted for age, sex, smoking history, alcohol consumption, TNM Stage, chemotherapy treatment, and surgery.

## Data Availability

The datasets used and/or analyzed during the current study are available from the corresponding author on reasonable request.

## Abbreviations

EORTC: European Organization for Research and Treatment of Cancer
HNSCC: Head and neck squamous cell carcinoma
LAMP1: Lysosomal-associated membrane protein 1
NF-*κ*B: Nuclear factor-kappa B
PBMC: Peripheral blood mononuclear cell
RTOG: Radiation therapy oncology group.
